# CNTNAP2 and Language Processing in Healthy Individuals as Measured with ERPs

**DOI:** 10.1371/journal.pone.0046995

**Published:** 2012-10-24

**Authors:** Miriam Kos, Danielle van den Brink, Tineke M. Snijders, Mark Rijpkema, Barbara Franke, Guillen Fernandez, Peter Hagoort

**Affiliations:** 1 Radboud University Nijmegen, Donders Institute for Brain, Cognition and Behaviour, Nijmegen, The Netherlands; 2 Radboud University Nijmegen, Behavioural Science Institute, Nijmegen, The Netherlands; 3 Department of Experimental Psychology, Helmholtz Institute, Utrecht University, Utrecht, The Netherlands; 4 Rudolf Magnus Institute of Neuroscience, Department of Child and Adolescent Psychiatry, University Medical Centre Utrecht, Utrecht, The Netherlands; 5 Department of Genetics, Radboud University Nijmegen Medical Centre, Nijmegen, The Netherlands; 6 Department of Psychiatry, Radboud University Nijmegen Medical Centre, Nijmegen, The Netherlands; 7 Department of Cognitive Neuroscience, Radboud University Nijmegen Medical Centre, Nijmegen, The Netherlands; 8 Max Planck Institute for Psycholinguistics, Nijmegen, The Netherlands; The University of Western Australia, Australia

## Abstract

The genetic *FOXP2-CNTNAP2* pathway has been shown to be involved in the language capacity. We investigated whether a common variant of *CNTNAP2* (rs7794745) is relevant for syntactic and semantic processing in the general population by using a visual sentence processing paradigm while recording ERPs in 49 healthy adults. While both AA homozygotes and T-carriers showed a standard N400 effect to semantic anomalies, the response to subject-verb agreement violations differed across genotype groups. T-carriers displayed an anterior negativity preceding the P600 effect, whereas for the AA group only a P600 effect was observed. These results provide another piece of evidence that the neuronal architecture of the human faculty of language is shaped differently by effects that are genetically determined.

## Introduction

People vary in their language abilities. There is compelling evidence that part of this variation has a genetic basis. Family and twin studies have revealed a large heritable component in language-related disorders. Moreover, heritable factors are also found to be responsible for part of the variance in healthy people’s linguistic abilities [1], [2]. Even though relatively little is known about the molecular genetic basis of language, several genes have been shown to play a role in language, such as the *FOXP2-CNTNAP2* pathway [2], [3]. The aim of the present study is to investigate whether a common variant of *CNTNAP2* (rs7794745) is relevant for syntactic and semantic processing in the general population.

*FOXP2 (forkhead box P2)* codes for a forkhead transcription factor and regulates the expression of other genes during development [4]. In vertebrates, *FoxP2* is widely expressed across the brain. More specifically, it is expressed in distributed circuits involving the cortex, basal ganglia, thalamus and cerebellum [5], [6]. Mutations in *FOXP2* cause severe but rare syndromic deficits in language comprehension and expression. These coincide with apraxic speech and orofacial praxis deficits including simultaneous and sequential movements, whereas other aspects of cognition and development are relatively spared (Developmental Verbal Dyspraxia; DVD [MIM 602081]) [7]–[11]. Brains of individuals carrying a mutation of *FOXP2* show subtle structural and functional abnormalities in language-related regions, such as the inferior frontal gyrus, superior temporal gyrus, cerebellum and striatum [12]–[16]. These results are consistent with behavioural evidence that *FOXP2* is associated with human language and speech. *FOXP2’s* role in vocal motor behaviour, however, is not restricted to humans, as *FoxP2* plays a role in vocal learning and motor sequencing in non-human species as well. Specifically, reduced functional levels of *FoxP2* protein have been found to impair vocalization and motor-skill learning in songbirds and mice respectively [5], [17], [18].

*FOXP2* regulates the expression of other genes, and one of its targets is the *CNTNAP2* gene [19]. *CNTNAP2* (*contactin associated protein-like 2*) encodes a protein belonging to the neurexin family [20] which is known to be involved in cell adhesion [5] and shows enriched expression in language-related circuits of the brain [21]. Several reports indicate a specific involvement of *CNTNAP2* in language development. It has been related to impaired speech development in Pitt-Hopkins-like syndrome involving intellectual disability [22], [23], to language regression in recessive symptomatic focal epilepsy [24], and to delays in language acquisition [25], [26], semantic-pragmatic skills [26] and speech [27] in autism. Furthermore, analyses of children with Specific Language Impairment (SLI) have associated *CNTNAP2* variants with reduced performance on indices of language ability such as nonword repetition [19], [28], expressive and receptive skills [19] as well as reading skills [28]. Recently, the observed association between *CNTNAP2* and nonword repetition was replicated in another language disorder, namely dyslexia [29] (but see [28]).

Importantly, *CNTNAP2* is not only associated with clinically distinct syndromes involving disrupted language. Recently, Whitehouse et al. [30] found that specific common genetic variants in the exon 13–15 region of *CNTNAP2*, previously linked to SLI [19], [29] and delayed language development in autism [25], are also related to the early stages of language development in children from the general population. Moreover, Whalley et al. [31], Snijders [32] and Folia et al. [33] found that another common single nucleotide polymorphism (SNP) of *CNTNAP2* (rs7794745), known to be involved in autism [34], is associated with functional brain measures related to language processing in healthy adults. Using magnetic resonance imaging (MRI) studies, these studies revealed differences in brain activation to sentence processing compared to the processing of word lists in right inferior frontal and left middle temporal cortex for two *CNTNAP2* rs7794745 genotype groups (AA vs T-carriers) [32]. A second study found differences between TT and A-carriers in the right middle temporal cortex during a sentence completion task [31]. Furthermore, differences in brain connectivity patterns between left inferior frontal cortex and left superior temporal cortex have been observed between AA and T-carriers as a function of the syntactic complexity of sentences (i.e. sentences containing word category ambiguities versus unambiguous sentences) [32]. This latter finding of *CNTNAP2* being associated with neurocognitive processing as a function of syntactic complexity has been confirmed by a magnetic encephalography (MEG) study using a similar experimental paradigm [32]. Finally, the two genotype groups (AA, T-carriers) of *CNTNAP2* rs7794745 also showed differences in behavioural as well as neuronal responses in language-related areas within an implicit artificial syntax learning study [33].

In sum, data from individuals with language-related disorders as well as healthy subjects are consistent with a role for *CNTNAP2* in language processing. Furthermore, four recent brain imaging studies (using MEG and fMRI) also suggest that the common rs7794745 variant of *CNTNAP2* is related to language or syntactic processing [31]–[33]. The goal of the current study was to further explore the effects of this variant on language or, more specifically, syntactic and semantic processing. As the rs7794745 variant of *CNTNAP2* is found to be most consistently involved in language processing in the general population [31]–[33], we selected this particular SNP of CNTNAP2 to investigate visual sentence processing using event-related brain potentials (ERPs) in healthy adults.

To examine syntactic processing we made use of a subject-verb agreement manipulation (e.g. The spoiled child **throws/throw** the toy on the floor.), known to elicit a positive ERP deflection (the P600) starting from around 600 ms after onset of a visually or auditorily presented word [35]–[38]. It is assumed that the P600 is a reflection of syntactic unification and that its amplitude is affected by competition between alternative unification options [39], [40]. Others suggest that the P600 reflects syntactic reanalysis or repair [41] or prefer a more general cognitive interpretation of the P600 effect, such as categorization or error monitoring [42], [43]. The P600 effect to syntactic manipulations is frequently preceded by (left or bilateral) anterior negativities occurring between 100 and 500 ms after word onset [37], [38], [44]–[46], reflecting a violation of the expectancy for a certain agreement feature [44] or the identification of word category and morphological information [41]. Others propose that these anterior negativities to agreement mismatches result from the failure to find a matching constituent to which the word can bind [40].

Our semantic manipulation consisted of sentences containing words that were semantically congruent or incongruent (e.g., Whipped cream tastes **sweet/anxious** and creamy). These semantically incongruent words have been shown to elicit a negative effect around 400 ms after the beginning of the word, known as the N400 effect [47]–[51]. While the N400 is generally considered to be an index of semantic processing, its precise functional interpretation is still under debate. The N400 effect is believed to reflect the pre-activation and unification of the meaning of a word into the overall meaning representation built upon the preceding language input [52]. Others propose that the N400 reflects the ease with which information can be accessed from long-term multimodal (i.e., semantic) memory [53], [54].

Summarizing, the *FOXP2-CNTNAP2* pathway seems to be implicated in language. In the current paper we looked into a common variant of *CNTNAP2* (rs7794745) identified in earlier brain imaging studies investigating syntactic ambiguities and artificial grammar learning. In the current study, we examine the relationship between this *CNTNAP2* rs7794745 variant to ERP responses sensitive to syntactic agreement and semantic processing, thus enabling us to see whether *CNTNAP2* rs7794745 is also involved in linguistic domains outside of syntax.

## Methods

### Ethics Statement

Written informed consent was obtained from all participants prior to measurement and the study received ethical approval from the local reviewing committee “CMO Arnhem Nijmegen” (CMO no 2001/095 and amendment “Imaging Human Cognition” 2006, 2008), in accordance with the local National law Research involving Human subjects Act, following the principles of the Declaration of Helsinki.

### Participants

In total, sixty Dutch native speakers of European descent participated in the experiments (26 male, mean age 21.3, range 18–30), 49 of whom were included in the final ERP analysis (24 males, mean age 21.3 years, range 18–30). Participants were recruited from the Donders Institute participant pool. All had normal or corrected-to-normal vision and were right-handed. None of the participants had any neurological or language impairment.

### Materials

#### Agreement manipulation

To examine syntactic agreement, we selected 80 Dutch sentence pairs from Hagoort et al. [35], where one sentence contained a number agreement violation between the subject and the verb and the other served as a correct control. These agreement violations are known to elicit a standard P600 effect [35]. The sentence pairs were identical with the exception of one word, which served as the critical word for the ERP analysis (printed in bold). In half of the cases the critical word was the verb of the sentence (e.g., The spoiled child **throws/throw** the toys on the floor), in the other half, the subject was the critical word (e.g., With an apple in the hand walk/walks the **sisters** to school; in Dutch the verb can appear in front of the subject, so the sentence with ‘walk’ is legal). The length of the sentences ranged from 5 to 14 words (mean 10.7 words, sd = 1.69).

#### Semantic manipulation

The experimental materials of the semantic manipulation consisted of 80 Dutch sentence pairs containing a semantic violation and a correct control. These sentence pairs had already been used in other experiments and are known to elicit an N400 effect [47]–[49]. Again, the experimental sentence pairs were identical with the exception of one word, which was the critical word for our analyses. Each pair consisted of a sentence that was semantically coherent (e.g., Whipped cream tastes **sweet** and creamy) and a sentence that contained a semantic anomaly (e.g., Whipped cream tastes **anxious** and creamy). The critical words were never in sentence-final position and were matched across conditions for word frequency, based on log lemma frequencies of the Dutch database CELEX [55] (semantically congruent = 2.96, semantically anomalous = 2.86), and length (semantically congruent = 5.69, semantically anomalous = 5.73). The length of the sentences ranged from 5 to 19 words (mean 12.7 words, sd = 3.0).

#### Other materials

In addition to the sentences of interest in this paper, participants also read a set of ambiguous relative clauses and a semantic-thematic manipulation [36] in one version of the experiment. The other version contained a set of complement clauses and a set of relative clauses [56]. Both versions contained 50 coherent items, which served as filler sentences. These coherent sentences were selected from the Dutch CLEF corpus [57]. In addition, we included 20 practice-items, which were similar to the experimental items.

The two different versions of the experiment, consisting of 434 sentences and 398 sentences respectively, were each mixed pseudo randomly. This was done in such a way that participants each got one version of an item, and that different versions were distributed equally across participants. Critical words were only used once in the critical position. The length of the sentences ranged from 5 to 19 words. The average length was 10.8 words (sd = 2.10).

### 2.3 Procedure

Participants were tested individually in a sound-attenuating booth. The booth was dimly lit (Fiber optic lights DMX 512 at 60%). Participants were seated in a comfortable chair and were told that the aim of the experiment was to investigate how people process sentences and that some of the sentences would be more difficult or strange than other sentences. Participants were informed that they were going to see a printed sentence that would be presented word-by-word in the middle of the computer screen, were instructed to read the sentences carefully and to attempt to understand them as well as possible. They were asked to try not to move or blink during the presentation of the sentence. No other task demands were imposed.

After a short practice session, trials were presented in five blocks of 15 min each, separated by rest periods of approximately 5 min. Halfway through every block there was an additional 30 s break. The viewing distance was approximately 110 cm. The first word of the sentence started with a capital letter and the rest of the words were presented in white lowercase ARIAL (23-point font size) against a dark background in the centre of a CFT 60 Hz monitor. Each word was presented for 300 ms followed by a blank screen for 300 ms, and the final word of the sentence ended with a period. After the final word an asterisk appeared for 2 s, indicating to the participants that they could blink and move their eyes, followed by a 1.2 s blank interval before the start of the next trial. Sentences were presented using Presentation software (Neurobehavioral systems, www.neuro-bs.com).

### Genetic Analysis

DNA was isolated from saliva, which was collected using the Oragene containers (DNA Genotek Inc., Kanata, Ontario, Canada) according to the protocol supplied by the manufacturer. DNA-isolation and genotyping were performed in a CCKL-accredited laboratory at the Department of Genetics of the Radboud University Nijmegen Medical Centre in Nijmegen. The *CNTNAP2* polymorphism (rs7794745, A>T) was genotyped using Taqman analysis (assay ID: rs7794745: Taqman assay C__2661558_10, reporter 1: VIC-A-allele, forward assay; Applied Biosystems, Nieuwerkerk a/d IJssel, The Netherlands). This particular SNP is located in the intron between exons 2 and 3 of the *CNTNAP2* gene. Genotyping was carried out in a volume of 10 µl containing 10 ng of genomic DNA, 5 µl of Taqman Mastermix (2x; Applied Biosystems), 0.125 µl of the Taqman assay and 3.875 µl of MilliQ. Amplification was performed by an initial denaturation at 95°C for 12 min, followed by 40 cycles of denaturation at 92°C for 15 s and annealing/extension at 60°C for 1 min. This was carried out on a 7500 Fast Real-Time PCR System, and genotypes were scored using the algorithm and software supplied by the manufacturer (Applied Biosystems). Generally, 5% blanks as well as duplicates were taken along as quality controls during genotyping.

Thirty-two participants were homozygous for the A allele (AA group), and twenty-eight participants were carrier of at least one T allele (AT/TT group: 20 AT, 8 TT). Testing for Hardy-Weinberg equilibrium did not show deviations from the expected distribution of genotypes (HWE, p = .11). For further analysis, carriers of at least one T allele, who have an increased risk for autism susceptibility [34], were grouped together and compared to carriers of the AA homozygous people, similar to the analyses performed by Snijders and Folia et al. [32], [33].

### EEG Recording and Analysis

The electroencephalography (EEG) was recorded from 28 cap-mounted Ag/AgCl electrodes (Easycap and Acticap). Four electrodes were placed over the standard 10% system midline sites Fz, FCz, Cz, and Pz. Eleven pairs were located over the standard lateral sites FP1/FP2, F7/F8, F3/F4, FC5/FC6, FC1/FC2, T7/T8, C3/C4, CP5/CP6, CP1/CP2, P7/P8, and O1/O2. Two electrodes were placed at the outer left and right canthi to monitor horizontal eye movements. Vertical eye movements were monitored using FP1 and an electrode placed below the left eye. An additional electrode was placed on the right mastoid bone. During measurement, all electrodes were referenced to the left mastoid. For the Easycap electrode impedances of the EEG- and electrooculographic (EOG) electrodes were kept below 5 and 10 kΩ respectively, for the Acticap electrode impedances were kept below 20 kΩ. Signals were recorded with a BrainAmp DC amplifier (Brain Products, Gilching, Germany), using a 125 Hz low-pass filter, a time constant of 10 s (0.016 Hz), and a 500 Hz sampling frequency. The software package Brain Vision Analyzer (Brain Products) was used to analyze the waveforms.

Offline, the EEG electrodes were rereferenced to the mean of the right and left mastoid and the EOG electrodes were converted into bipolar horizontal and vertical EOG signals. A 30 Hz, 12 dB low-pass Hanning filter was applied. Subsequently, the critical words were segmented using a window which started 200 ms before and ended 1500 ms after the critical word. After baseline correcting to the 200 ms interval before the critical word, segments were semi-automatically screened for eye movements, electrode drifting, amplifier blocking and electromyographic (EMG) artefacts using a 75 µVolt criterion. Segments containing such artefacts were rejected (12.1% overall) with no asymmetry across conditions (range of segments which were included in the average: syntactically congruent: 31–40, syntactically anomalous: 30–40; semantically congruent: 26–40, semantically anomalous: 30–40). The remaining EEG segments were averaged per participant and per condition. Ten participants were excluded from the analysis due to an excessive number of artefacts in the EEG signal and one participant was excluded due to technical problems during the measurement, leaving 49 participants for subsequent analysis (24 males, mean age 21.3 years, range 18–30; characteristics per genotype group are displayed in Table 1).

**Table 1:** Genotype group characteristics.

Genotype group	Number	Mean age (Range)	Gender
AA	26	22.0 (18–30)	11 males
AT-TT	23	20.5 (18–24)	12 males

With respect to the syntactic manipulation, a latency window between 150 and 550 ms after onset of the critical word was selected to test for (early) anterior negativities. This time window was based on visual inspection. For assessment of the P600 effect a standard 600–1000 ms latency window was applied. A standard latency window of 300 to 550 ms after onset of the critical word was used to compute the mean amplitude of the N400 component. The effects were evaluated in repeated-measures analyses of variance (ANOVA) involving the between-subject factor Genotype (AA, AT/TT) and the within-subject factors syntactic or semantic Congruency (congruent, incongruent) and Site, which consisted of two levels Anterior (F7, F3, Fz, F4, F8, FC5, FC1, FCz, FC2, FC6) and Posterior (Cz, CP5, CP1, CP2, CP6, P7, P3, Pz, P4, P8). Interactions with the factors Genotype and/or Site were followed by separate Genotype and Site analyses.

## Results

### Agreement Manipulation

**Figure 1:**
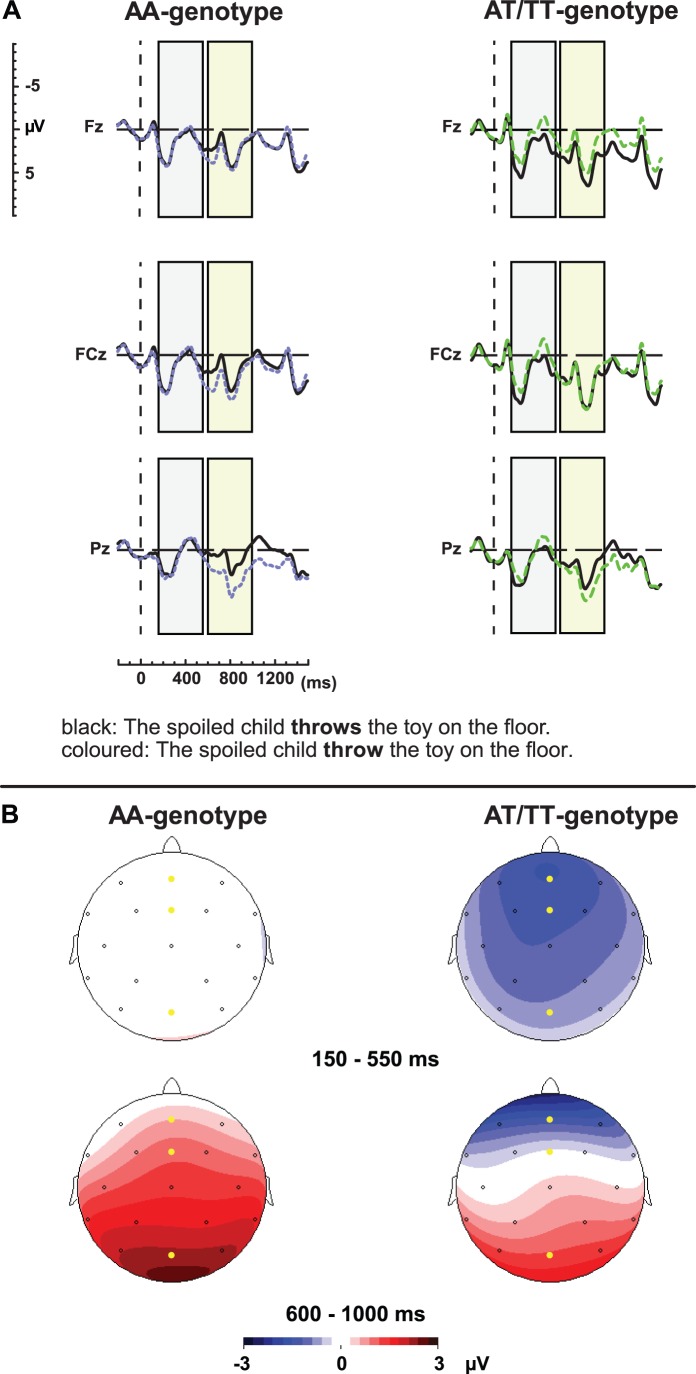
*CNTNAP2* and syntactic manipulation. a. ERP waveforms elicited by the agreement violations (dotted, coloured line) and their correct controls (solid, black line) for the AA and AT/TT genotype groups for electrodes Fz, FCz, and Pz. The left, grey block and right, green block indicate the latency windows used for analysis for the anterior negativity and P600 respectively. In this and the following figure the waveforms are time-locked to the onset of the critical word and negative voltage is plotted upward. An 8 Hz low-pass filter has been applied for illustrative purposes. **b.** Scalp distribution of the effect elicited by the agreement manipulation between 150 and 550, as well as 600 and 1000 ms after critical word onset. In this and the following figure the electrodes for which the waveforms are displayed have been highlighted.

Figure 1a shows the average waveforms of the agreement violations and their correct controls, for the AA and AT/TT genotype groups. Figure 1b depicts the topographical distribution of the agreement effect between 150 and 550 ms and 600 and 1000 ms of the two genotype groups. Even though both groups show the typically posteriorly distributed P600 effect, only the AT/TT group showed the earlier, negative-going effect maximal at anterior sites.

### 150­550 ms

The repeated measures ANOVA in the 150 to 550 ms latency window revealed a main effect of Syntactic Congruency (F(1,47) = 4.81, MSE = 19.18, p<.05). There was a significant interaction between Congruency and Genotype (F(1,47) = 4.12, MSE = 19.18, p<.05; effect size = 0.56), the interaction between Congruency, Site and Genotype was not significant (p>.05), nor was the interaction between Congruency and Site (p>.05).

Post-hoc analyses for Congruency×Genotype interaction revealed no effect in the 150 to 550 ms time window for the AA-group (p>.05), whereas the AT/TT group exhibited a significant negativity (F(1,22) = 6.42, MSE = 25.10, p<.05; see Figure 1).

### 600–1000 ms

In the 600 to 1000 ms latency window a main effect of Syntactic Congruency was observed (F(1,47) = 5.12, MSE = 39.63, p<.05). Even though the topographical distributions for the two genotype groups (Figure 1b) seem slightly different, there were no significant interactions involving the factor Genotype (Congruency×Genotype: p>.05; Congruency×Genotype×Site: p>.05), indicating that both genotype groups elicited a P600 effect (Figure 1). The interaction between Congruency and Site was significant (F(1,47) = 39.92, MSE = 7.59, p<.001), revealing the typical posterior distribution of the P600 effect (anterior: Congruency: p>.05; posterior: Congruency: F(1,48) = 22.88, MSE = 22.61, p<.001).

On the basis of visual inspection it seemed that the onset of the P600 was earlier for the AA-group compared to the T-carriers. For this reason we performed additional analyses within 600–800 and 800–1000 latency windows. While we did not observe significant interactions with genotype group within the latter latency window (Congruency×Genotype: p>.05, Congruency×Genotype×Site: p>.05), we did observe a (marginally significant) Congruency×Genotype interaction in the earlier (600 and 800 ms) time window (F(1,47) = 4.03, MSe = 46.38, p = .05; Congruency×Genotype×Site: p>.05). Post-hoc tests per genotype group revealed that only the AA-group showed a positive effect where the T-carriers did not (AA-group: F(1,25) = 21.27, MSE = 22.17, p<.001; T-carriers: p>.05).

### Semantic Manipulation

**Figure 2:**
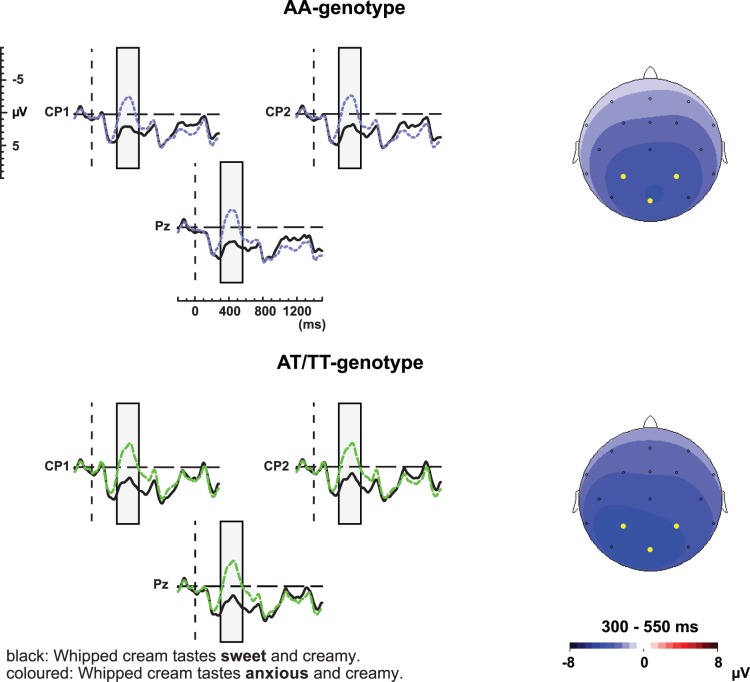
*CNTNAP2* and semantic manipulation. ERP waveforms elicited by the semantic anomalies (dotted, coloured line) and their correct controls (solid, black line) for the AA and AT/TT genotype groups for electrodes CP1, CP2, and Pz. The grey block indicates the latency window used for analyses of the N400 effect. The left panel of this figure depicts the scalp distribution of the effect elicited by the semantic manipulation between 300 and 550 ms after critical word onset.

Figure 2 depicts the average waveforms and concomitant topographical distribution of the semantic manipulation within the N400 time window for the AA and AT/TT genotype groups. In both groups the semantic anomalies elicited a clear N400 effect.

### 300–550 ms

Analyses between 300 and 550 ms revealed a significant effect of semantic Congruency (F(1,47) = 81.13, MSE = 45.16, p<.001). There were no significant interactions with the between-subject factor Genotype (Congruency×Genotype: p>.05; Congruency×Site×Genotype: p>.05), denoting that both genotype groups display a comparable N400 effect (Figure 2). We found a significant interaction between Congruency and Site (F(1,47) = 29.93, MSE = 6.45, p<.001). Post-hoc analyses for Site demonstrated significant effects for both anterior (F(1,48) = 39.34, MSE = 27.19, p<.001) and posterior electrodes (F(1,48) = 111.14, MSE = 24.67, p<.001).

## Discussion

In this paper we show that a common variant of *CNTNAP2* (rs7794745) is relevant for sentence processing as measured with ERPs. The brain response to syntactic number agreement violations was different for the two genotype groups (AA vs AT/TT) of this variant. While in both genotype groups the agreement violations elicited a P600 effect, only carriers of the T-allele exhibited an anterior negativity preceding the P600 effect. In addition, the P600 effect of the T-carriers seemed to have a later onset compared to the AA-group. However, we cannot exclude the possibility that this difference results from component overlap with the negativity between 150–550 ms observed for the T-carriers, therefore we refrain from functionally interpreting this difference. In contrast to the syntactic manipulation revealing clear neurocognitive processing differences between the *CNTNAP2* genotype groups, these groups did not show any processing differences with respect to the semantic manipulation, as both groups displayed a standard N400 effect to semantic anomalies.

These results are in line with earlier findings that suggest that the *FOXP2-CNTNAP2* pathway is implicated in language. Previous results have shown that mutations on *FOXP2* cause syndromic language and speech deficits [7]–[11]. Furthermore, *CNTNAP2*, one of the SNPs whose expression is regulated by *FOXP2,* is associated with impairments of language development in several syndromes, such as autism [25]–[27] and SLI [19], [28]. Recently, it has also been found that *CNTNAP2* is associated with language development in the general population [30].

The current findings are consistent with four recent brain imaging studies showing that the common variant rs7794745 of *CNTNAP2* is related to language processing in the general population [31]–[33]. In these studies differences across genotypes of this variant were observed with respect to brain activation for the processing or completion of sentences in right inferior frontal and left and right middle temporal cortex [31], [32]. Furthermore, brain connectivity patterns between left inferior cortex and left superior temporal cortex, as well as event-related fields over left temporal regions differed between *CNTNAP2* groups as a function of syntactic complexity [32]. In addition to these sentence processing measures, an artificial syntax learning paradigm Folia et al. [33] revealed differential brain responses in left inferior frontal cortex –in addition to the left frontopolar region- between *CNTNAP2* groups, with the AA-group showing larger activation compared to the T-carriers. Finally, behavioural results of this study showed that T-carriers acquired structural knowledge in a more efficient way compared to the AA-group, with less reliance on irrelevant, familiarity features of the surface sequences (local subsequence familiarity).

In sum, in those studies with strongly controlled language processing, it is observed that genotype differences found for this common variant of *CNTNAP2* in the general adult population pertain primarily to syntactic processes. Interestingly, *CNTNAP2* usually has been linked to broader domains of language development or capacity, comprising semantics, syntax and phonology (e.g. [19], [26], [30]). With the observation that this common variant of *CNTNAP2* is relevant largely for syntactic processes, we do not claim that this SNP is syntax-specific. Rather, this pattern of findings could suggest that this SNP is associated with the development of, or communication between those brain areas that are especially relevant for syntactic processing. Further research is necessary to see whether this common variant of rs7794745 is relevant for other or broader language domains as well.

How can we interpret the differential ERP pattern observed for the number agreement violations, with only T-carriers displaying an anterior negativity, in light of the previous findings for this common variant of *CNTNAP2* [31]–[33]? With respect to the processing of number agreement violations, it is known that its neural basis lies, amongst others, in the left inferior frontal gyrus (BA 44 and 45) and superior temporal gyrus [58], [59]. Additionally, areas assumed to underlie (early) anterior negativities are the superior temporal gyrus, middle temporal gyrus and left inferior frontal gyrus [41]. Hence, the areas known to be differentially functionally connected or activated for the two genotype groups of *CNTNAP2* in the previous brain imaging studies [31]–[33] overlap with the areas known to be involved in the processing of subject-verb agreement. Furthermore, the artificial syntax learning study showed that T-carriers seemed to be more sensitive to structural cues, while the AA-group relied more on ineffective surface properties of the sequences. Relating these observations to the knowledge that anterior negativities have been functionally linked to the processing of morphological features [40], [41], [44], a tentative explanation is that T-carriers, who show an anterior negativity, focus more on these specific grammatical features of words compared to the AA-group. As it is known that T-carriers have an increased susceptibility for autism, it would also be interesting to link our ERP findings to autism. Unfortunately, we are not aware of studies investigating syntactic processing by means of ERPs in autism. However, differences for autism with respect to language related ERPs have been reported before [60], [61].

In conclusion, the current study demonstrates an association of a common genetic polymorphism of *CNTNAP2* (rs7794745) with individual variation in neurocognitive response to a syntactic manipulation. While both genotype groups showed a P600 effect to number-agreement violations, only T-carriers displayed an anterior negativity preceding this P600 effect. These results provide another piece of evidence that the neuronal architecture of the human faculty of language is shaped differently by effects that are genetically determined.
